# Framing effects on bribery behaviour: experimental evidence from China and Uganda

**DOI:** 10.1007/s40881-018-0049-2

**Published:** 2018-06-04

**Authors:** Alessio Gaggero, Simon Appleton, Lina Song

**Affiliations:** 1 Nottingham University Business School, Nottingham, UK; 2 School of Economics, University of Nottingham, Ningbo, China

**Keywords:** Framing, Bribery behaviour, C91, D73, D91

## Abstract

In this study we investigate the effect of framing on bribery behaviour. To do this, we replicate Barr and Serra (Exp Econ, 12(4):488–503, (2009) and carry out a simple one-shot bribery game that mimics corruption. In one treatment, we presented the experiment in a framed version, in which wording was embedded with social context; in the other, we removed the social context and presented the game in a neutral manner. The contribution of this paper is that it offers a comparison of framing effects in two highly corrupt countries: China and Uganda. Our results provide evidence of strong and significant framing effects for Uganda, but not for China.

## Introduction and motivation

In the last decades, there has been increasing interest in the effects of framing on decision making and experimental tasks. In particular, recent studies have reported the existence of significant framing effects in prisoner’s dilemma games (Ross and Ward, 1996; Liberman et al. 2004), public goods games (Andreoni 1995; Cookson 2000; Rege and Telle 2004), and dictator games (Eckel and Grossman 1996; Branas-Garza 2007). There is little consensus among economists, however, on the existence of framing effects on bribery behaviour.

Abbink and Hennig-Schmidt (2006, AHS hereafter) first investigate whether, and to what extent, presenting a bribery game framed as a (repeated) corrupt exchange between a firm and a public official, as opposed to the same game framed neutrally and in abstract terms, would reduce bribery behaviour.^1^ In the study, the authors find no evidence that loading the context of the instructions with an immoral ethical preconception has an effect on bribery behaviour, for a sample of students at the University of Bonn.

As argued by Barr and Serra (2009), however, the obtained null results may be driven by either artificiality, in that participants did not identify themselves with either being a firm or a public official, or by the fact that the original AHS game was repeated for thirty rounds with fixed matching and hence also involved feelings of trust and reciprocity.^2^ Barr and Serra (2009) tackle these potential issues by designing a simple one-shot petty corruption experiment, in which participants interact with each other as ‘citizens’ and ‘officials.’ The main advantages of their setting are that (1) all students are citizens and, consequently, more likely to identify themselves with the frame adopted, and (2) in a one-shot game, feelings such as trust and reciprocity are negligible. Using a sample of students at the University of Oxford, the authors do find evidence of framing effects, and successfully show that the existence of a framing effect depends on the specific experimental design employed as well as the degree of artificiality of the corruption frame applied.

In this study, we contribute to the literature by replicating the Barr and Serra (2009) petty corruption game in two highly corrupt countries, China and Uganda, currently ranked 77th and 151st out of 176 respectively in the Corruption Perception Index. In 2012, China initiated the largest campaign against graft and corruption ever made in the history of its Communist Party, leading to the indictment of over 120 high-ranking officials and 100,000 individuals. The latter, although its corruption level keeps worsening, still has not embraced any official anti-corruption campaign. Our results provide evidence of strong and significant framing effects for Uganda, but not for China.

## Experimental design

The game includes 15 players. We randomly assigned players to three roles: private citizens, public officials, and other members of society (henceforth, ‘citizens’, ‘officials’, and ‘members of society’).

Each citizen is randomly matched with an official, and gets an initial endowment of $$ I_{C} $$. He has to decide whether to offer a bribe to the matched official for a corrupt service, for which he will obtain a benefit of $$ V $$. Let $$ B $$ be a dummy variable that equals unity if the citizen chooses to offer a bribe, and let $$ b \in \{ 1,2, \ldots ,20\} $$ denote the amount of the bribe offered. Notice, if the citizen decides to offer a bribe to the official, he will pay a (deterministic) cost, denoted $$ K, $$ which reflects the expected cost of getting caught and punished.

Each official receives an initial endowment of $I_{O}$. He has to decide whether or not to accept the bribe offered by the matched citizen. Let $A$ be a dummy variable which equals one if the official decides to accept the bribe. Similar to above, if the official accepts the bribe, he will also incur a deterministic loss,$K$, reflecting the expected cost of being caught.

Finally, each member of society receives an initial endowment of $I_{M}$. They are a passive group, and as such do not make any decisions in the game. They will, however, incur a loss of endowment, $h$, each time a citizen-official pair chooses to act corruptly.

Let $$ Y_{C} , Y_{O} , $$ and $$ Y_{M} $$ represent the final payoffs of the citizens, officials, and members of society, respectively, at the end of the bribery game. Thus, the payoff schedule for the three types of players can be written in the following compact form:

1 $$ Y_{\text{C}} = \left\{ {\begin{array}{*{20}l} {I_{\text{C}} } \hfill & { \quad {\text{if}}\;B = 0 } \hfill \\ {I_{\text{C}} - K} \hfill & { \quad {\text{if}}\;B = 1\; {\text{and}}\;A = 0} \hfill \\ {I_{\text{C}} + V - K - b} \hfill & {\quad {\text{if}}\;B = 1\;{\text{and}}\;A = 1,} \hfill \\ \end{array} } \right. $$

2 $$ Y_{\text{O}} = \left\{ {\begin{array}{*{20}l} {I_{\text{O}} } \hfill & {\quad {\text{if}}\;A = 0} \hfill \\ {I_{\text{O}} + b - K} \hfill & {\quad {\text{if}}\;A = 1} \hfill \\ \end{array} } \right. $$

3 $$ Y_{\text{M}} = I_{\text{M}} - N\, \cdot \,h, $$

where $N \in \{ 0, 1, 2, 3, 4, 5\}$ represents the number of citizen-officials pairs who successfully exchange bribes. Following the original paper, we set $I_{\text{C}} = I_{\text{O}} = 35$ money units and $I_{\text{M}} = 25$ money units.^3^ Further, we set $V = 20$, $h = 4$, and $K = 5$. A total of 54 experimental sessions were conducted, 27 in each country.^4^ Every session involved 15 players, with the exception of two sessions in China, in which we only managed to recruit 9 and 12 players, respectively. Table 1 presents demographic features of the sample of students, and reports that participants in China were younger and there was a higher proportion of females.^5^ The sessions were conducted in various teaching and seminar rooms, depending on availability, and carried out with pen and paper. Participants were randomly allocated to their role, and at no time knew which player they were matched with. Besides the instructions, on each desk, participants found tables describing the payoff schedule of the game, and an envelope containing a specially designed form for players to express their preferences. In particular, citizens had to state the amount, if any, they wanted to offer to their matched official; officials, instead, had to state whether they would accept or reject each one of the possible bribes, $b \in \{ 1,2, \ldots ,20\}$. This allows us to identify officials who would reject all of the possible bribes, as well as the minimum amount offered for the bribe to be accepted. Once all the forms were filled, envelopes were collected and payoffs calculated.

**Table 1:** Summary statistics

	(1) China	(2) Uganda	(3) *p* value
Female	0.77 (0.424)	0.34 (0.474)	0.000
Years of age	21.45 (1.952)	23.87 (3.573)	0.000
Father years of schooling	11.82 (2.867)	10.82 (3.864)	0.000
Mother years of schooling	10.95 (3.326)	9.13 (4.198)	0.000
Observations	396	405	801

The table presents summary statistics of the experimental sample, comparing China and Uganda. Standard deviations in parenthesis

We had two treatments of the game. In the framed treatment, each role and action was embedded in the social context, as explained above. In the neutral treatment, however, we removed the social context and presented the game in an abstract manner. Specifically, player roles went from citizen, official, and member of society, to player A, player B, and player C, respectively; and the word “bribe” was replaced with the word “offer”. In each country, we randomly drew 14 sessions to be presented in the framed version, and the remaining in the neutral one.

Before presenting our result, it is important to mention the power of this replication. Accordingly, we follow the guidelines of Nikiforakis and Slonim (2015) and Drichoutis et al. (2015), and implement a power calculation for our study to find whether or not there is an effect statistically significant at the 5% level. Given our sample size, and the observed means and standard deviations in the original study of Barr and Serra (2009), the power of our experiment is 99%.^6^

## Results

Table 2 reports the effect of framing on bribery behaviour. Specifically, in columns (1) and (2) we compare the behaviour under the neutral and framed treatments for the Chinese sample, and in column (3) we report the *p* value of a t-test which compares the means of the two groups. Similarly, in columns (4), (5), and (6), we report the same for the participants from Uganda. The table shows that framing had virtually no effect on the behaviour of participants in China. Whether the game was played in the neutral or in the framed version, an average of 4.6 successful bribes per session occurred, that is, citizens’ offers were successfully accepted by the randomly paired officials. The table shows that only a small proportion of Chinese students either did not offer any bribes (8%), or did not accept any possible bribes (2%). Similarly, framing had no effect on the conditional amount of bribes offered, nor accepted.

**Table 2:** Descriptive analysis

	China			Uganda		
	(1) Neutral	(2) Framed	(3) *p* value	(4) Neutral	(5) Framed	(6) *p* value
Successful bribes per session private Citizen	4.62 (0.045)	4.61 (0.040)	0.715	4.46 (0.045)	3.42 (0.072)	0.000
Offered no bribe	0.08 (0.267)	0.06 (0.239)	0.715	0.11 (0.312)	0.31 (0.468)	0.003
Mean bribe offered	7.67 (3.439)	8.21 (3.038)	0.361	6.66 (6.192)	5.77 (4.660)	0.416
Public official						
No acceptable bribes	0.02 (0.124)	0.04 (0.207)	0.336	0.05 (0.211)	0.20 (0.403)	0.007
Conditional mean acceptable offer	11.67 (3.904)	11.55 (3.433)	0.856	13.23 (4.107)	12.64 (4.201)	0.448
Observations	131	135	266	130	140	270

The table compares bribing behaviour of participants who played the bribery game in the framed and in the neutral treatment. Standard deviations in parenthesis

Conversely, framing had a strong and significant effect in Uganda. Specifically, students who played the bribery game in the framed form were significantly less likely to either offer or accept any bribe, and consequently, the number of successful bribes in framed sessions were significantly lower than in the neutral ones. The table, moreover, shows that while framing has a strong effect on the propensity to either offer or accept a bribe, it does not have an effect on the conditional amount offered nor on the conditional amount accepted.

In Tables 3 and 4, we check the robustness of our results in a regression framework that allows us to control for a set of potentially confounding variables, as well as allowing for interdependence within sessions by clustering. Specifically, Table 3 reports framing effects on the citizens, and in Table 4 we report estimated coefficients for the officials. The results confirm our descriptive analysis and show strong and significant framing effects for the sample from Uganda, and no significant effect for China. Further, in order to directly test whether there is a significant differential framing effect between the two countries, in Table 5, we conduct a regression analysis on the pooled data in which we include an interaction term combining the framing dummy variable together with the Uganda dummy variable. The table confirms a statistically significant differential framing effect for the private citizens in Uganda with respect to the private citizens in China, but reports no differential framing effect for the public officials between the two countries.

**Table 3:** Framing effects for private citizens

	China		Uganda	
	(1) Offered no bribe	(2) Mean bribe offered	(3) Offered no bribe	(4) Mean bribe offered
Framed [0, 1]	− 0.067 (0.367)	0.524 (0.599)	0.727*** (0.266)	− 0.784 (1.081)
Covariates				
Female	− 0.186 (0.431)	− 0.387 (0.727)	− 0.235 (0.277)	− 2.814** (1.144)
Years of age	− 0.164 (0.142)	− 0.010 (0.146)	− 0.025 (0.043)	0.028 (0.151)
Beijing	0.107 (0.557)	− 0.702 (0.816)		
Chengdu	0.450 (0.543)	0.683 (0.835)		
Kampala			− 0.016 (0.312)	1.269 (1.310)
Constant	1.886 (3.137)	8.216** (3.356)	− 0.524 (1.194)	6.057 (4.297)
Observations	132	124	133	104

The table presents regression estimates of the effect of framing on corruption behaviour. Probit models were estimated if the dependent variable was dichotomous [0, 1]. Standard errors in parenthesis are clustered at the session level * p < 0.1, ** p < 0.05, *** p < 0.01

**Table 4:** Framing effects for public officials

	China		Uganda	
	(1) Accepted no bribe	(2) Minimum acceptable bribe	(3) Accepted no bribe	(4) Minimum acceptable bribe
Framed [0, 1]	0.473 (0.567)	0.230 (0.641)	0.947*** (0.335)	-0.071 (0.794)
Covariates				
Female	− 1.034 (0.716)	− 0.438 (0.831)	− 0.214 (0.340)	− 0.469 (0.839)
Years of age	− 0.173 (0.131)	− 0.617*** (0.177)	− 0.058 (0.077)	0.119 (0.128)
Beijing	4.232 (702.133)	− 1.730* (0.876)		
Chengdu	5.096 (702.133)	− 1.072 (0.918)		
Kampala			0.563 (0.454)	1.930* (0.983)
Constant	− 2.421 (702.138)	26.115*** (4.028)	− 0.781 (2.009)	9.034** (3.577)
Observations	132	128	132	115

The table presents regression estimates of the effect of framing on corruption behaviour. Probit models were estimated if the dependent variable was dichotomous [0, 1]. Standard errors in parenthesis are clustered at the session level * p < 0.1, ** p < 0.05, *** p < 0.01

**Table 5:** Framing effects

	Private citizen				Public official:			
	(1) Offered no bribe	(2) Offered no bribe	(3) Mean bribe offered	(4) Mean bribe offered	(5) Accepted no bribe	(6) Accepted no bribe	(7) Minimum acceptable bribe	(8) Minimum acceptable bribe
Framed [0, 1]	− 0.123 (0.320)	− 0.087 (0.343)	0.534 (0.481)	0.363 (0.499)	0.455 (0.508)	0.305 (0.468)	− 0.118 (0.687)	0.038 (0.673)
Uganda	0.195 (0.309)	0.344 (0.546)	− 1.017 (0.997)	− 3.085*** (1.032)	0.477 (0.510)	3.084*** (0.430)	1.554** (0.735)	− 1.013 (1.085)
Framed* Uganda	0.878** (0.406)	0.841** (0.412)	− 1.419 (1.358)	− 1.008 (1.308)	0.386 (0.629)	0.676 (0.594)	− 0.465 (1.064)	− 0.179 (1.005)
Covariates								
Female		− 0.157 (0.229)		− 1.756*** (0.618)		− 0.303 (0.272)		− 0.216 (0.589)
Year of Enrolment		− 0.051 (0.133)		− 0.285 (0.328)		− 0.183 (0.156)		− 0.231 (0.254)
Beijing		0.196 (0.551)		− 0.385 (0.614)		3.513*** (0.618)		− 1.323 (0.826)
Chengdu		0.427 (0.476)		1.018 (0.725)		4.094*** (0.504)		− 0.953 (0.971)
Kampala		0.085 (0.301)		1.391 (1.010)		0.753** (0.315)		1.537* (0.778)
Constant	− 1.434*** (0.242)	− 1.453** (0.701)	7.672*** (0.414)	9.991*** (1.516)	− 2.160*** (0.385)	− 4.880*** (0.355)	11.672*** (0.513)	13.625*** (1.274)
Observations	266	266	229	229	264	264	243	243

The table presents regression estimates of the effect of framing on corruption behaviour. Probit models were estimated if the dependent variable was dichotomous [0, 1]. Standard errors in parenthesis are clustered at the session level * p < 0.1, ** p < 0.05, *** p < 0.01

## Discussion and conclusion

In this paper, we replicate Barr and Serra (2009) one-shot petty corruption game, and compare framing effects between two countries with a high level of corruption: China and Uganda. We find strong and significant framing effects for the participants from Uganda. Specifically, students exposed to the framed version of the petty corruption game were significantly less likely to offer, as well as accept, any bribe. On the contrary, we do not find any significant effects of framing on bribery behaviour in China.

The results for Uganda are consistent with those obtained by Barr and Serra (2009); however, this is not the case for China, suggesting other factors might be causing the differences in framing effects between the two countries. Following Cooper et al. (1999) and Barr and Serra (2010), the magnitude of the framing effect may change depending on the degree participants have been exposed to a corrupt environment, as well as on the social norms and values prevailing in a certain society. For example, it is likely that in a society with a higher exposure to corruption, in which people engage in bribery in their everyday life, individuals would be less responsive to the corruption frame. As both China and Uganda have high levels of corruption, it is not obvious that differences in the degree of corruption in the two countries explain our result. Indeed, our results are somewhat paradoxical: Transparency International’s data (based on perceptions by elites and business people) suggest Uganda has higher levels of corruption than China. Uganda certainly lacks China’s fierce official anti-corruption drive. However, our Ugandan subjects are more sensitive to framing the experiment in terms of corruption than their Chinese counterparts. Nor is there clear evidence that corruption is more socially acceptable in China than Uganda. Indeed, in the World Values Survey, Chinese citizens were less likely to agree that “it was justifiable that someone accepts a bribe in the course of their duties”. On scale of 1 = never justifiable to 10 = always justifiable, China averaged 1.20 in 1995 while Uganda averaged 2.14 in 2001—the latest years available (Inglehart et al. 2014; Gatti et al. 2003).

Further research is necessary to explain our results. This may be a case where there is a disconnect between the attitudes people espouse in public opinion surveys and their behaviour in private games. One may disapprove of something in public—particularly when done by others—but engage in it personally in private. China has a culture of Guanxi (or personal relations) in business that can involve gift giving which potentially strays into corruption (Zhan 2012). For example, the government has recently cracked down on lavish banquets by public officials. A 2014 Pew survey of public attitudes found 38% of Chinese respondents thought bribery was important for getting ahead—the highest proportion in all the countries in the world and ahead of the 15% average for the African countries in the sample (which included Uganda, but the country specific figure was not available, Gao, 2014). Potentially related to this, corruption has sometimes been seen by economists as “greasing the wheels” of economic development in China by substituting for private property rights and the rule of law. For example, Qian (2003) argues that the incentivising cadres was the key to successful reform in China: local officials would encourage the development of publicly or collectively owned enterprises because they received a private share of the benefits (sometimes through corruption). Conversely, economists studying Uganda (and indeed the rest of Africa), tend to view corruption unequivocally a barrier to economic development, raising the costs of doing business, undermining government services, private property rights and the rule of law (Godfrey and Yu 2014). Future research to understand better private behaviour regarding corruption may be very important for policy, as anti-graft campaigns may not be successful if citizens do not adhere to the expected norms against corruption.

## Appendix

See Tables 6 and 7.

**Table 6:** Comparison

	China			Uganda			Barr and Serra		
	(1) Neutral	(2) Framed	(3) Diff.	(4) Neutral	(5) Framed	(6) Diff.	(7) Neutral	(8) Framed	(9) Diff.
No bribe offered	0.08 (0.267)	0.06 (0.239)	0.02 (0.044)	0.11 (0.312)	0.31 (0.468)	− 0.21 (0.069)	0.10 (0.305)	0.40 (0.497)	− 0.30 (0.104)
No acceptable bribes	0.02 (0.124)	0.04 (0.207)	− 0.03 (0.030)	0.05 (0.211)	0.20 (0.403)	− 0.15 (0.056)	0.10 (0.305)	0.26 (0.443)	− 0.16 (0.096)
Observations	131	135	266	130	140	270	30	35	65

The table compares results of our experimental sample with those of Barr and Serra (2009). Standard deviations in parenthesis

**Table 7:** Framing effects

	Private citizen				Public official			
	(1) Offered no bribe	(2) Offered no bribe	(3) Mean bribe offered	(4) Mean bribe offered	(5) Accepted no bribe	(6) Accepted no bribe	(7) Minimum acceptable bribe	(8) Minimum acceptable bribe
Framed [0, 1]	0.59.3* (0.307)	0.616** (0.314)	− 0.235 (1.236)	− 0.476 (1.251)	0.814** (0.324)	0.815** (0.355)	− 0.683 (0.780)	− 0.407 (0.694)
Female	− 0.263 (0.334)	− 0.098 (0.376)	− 0.990 (0.787)	− 1.777** (0.852)	− 0.498 (0.368)	− 0.332 (0.479)	− 1.288 (0.808)	− 0.394 (0.816)
Framed* female	− 0.249 (0.401)	− 0.207 (0.392)	0.415 (1.309)	0.336 (1.281)	− 0.087 (0.439)	0.014 (0.458)	0.673 (1.048)	0.488 (0.954)
Covariates								
Year of Enrolment		− 0.108 (0.118)		0.044 (0.309)		− 0.204 (0.136)		− 0.110 (0.226)
Beijing		− 0.411 (0.470)		1.437* (0.731)		0.079 (0.556)		− 0.738 (0.785)
Chengdu		− 0.209 (0.339)		2.799*** (0.804)		0.604 (0.505)		− 0.426 (0.920)
Kampala		0.216 (0.295)		0.078 (1.049)		0.820*** (0.303)		1.168* (0.684)
Constant	− 1.231*** (0.246)	− 0.991* (0.553)	7.714*** (0.843)	7.246*** (1.435)	− 1.668*** (0.277)	− 1.697*** (0.493)	13.150*** (0.570)	12.763*** (0.900)
Observations	266	266	229	229	264	264	243	243

The table presents regression estimates of the effect of framing on corruption behaviour. Probit models were estimated if the dependent variable was dichotomous [0, 1]. Standard errors in parenthesis are clustered at the session level ** p* < 0.1, *** p* < 0.05, **** p* < 0.01

## Footnotes

1 The game was first designed by Abbink et al. (2002).

2 See Bardsley (2005) for a discussion of artificiality in experimental economics.

3 This difference in initial endowments reflects the fact that other members of the society do not usually have the means to engage in corrupt behaviour.

4 In order to increase the representativeness of our samples, experiments were carried out in Kampala and Jinja, in Uganda, and Beijing, Ningbo, and Chengdu in China.

5 It is important to emphasise that regression analysis can only partially account for this important difference in composition of men and women across countries. In order to dispel potential issues, in Table 7 in the “Appendix”, we check for the possibility of a composition effect, but this does not seem to be the case.

6 Table 6 in the “Appendix” compares our experimental results with those of Barr and Serra (2009).
